# Supplementary material for: Mediating roles of character traits and parenting in the relationship between maternal effortful control and children’s conduct problems
Source: PeerJ. 2023 Apr 10;11:e15211. doi: 10.7717/peerj.15211 (PMC10100806; doi:10.7717/peerj.15211)
Supplement: Supplemental Information 3 [file peerj-11-15211-s003.docx]

Test Instrument Permissions

- CCNES

The use is in accordance with a published license: [Coping with Children's Negative Emotions Scale (ccnes.org)](https://ccnes.org/)

- TCI-R

Prof. Zohar is the Israeli developer of the TCI-R and Prof. Cloninger has attached is consent.


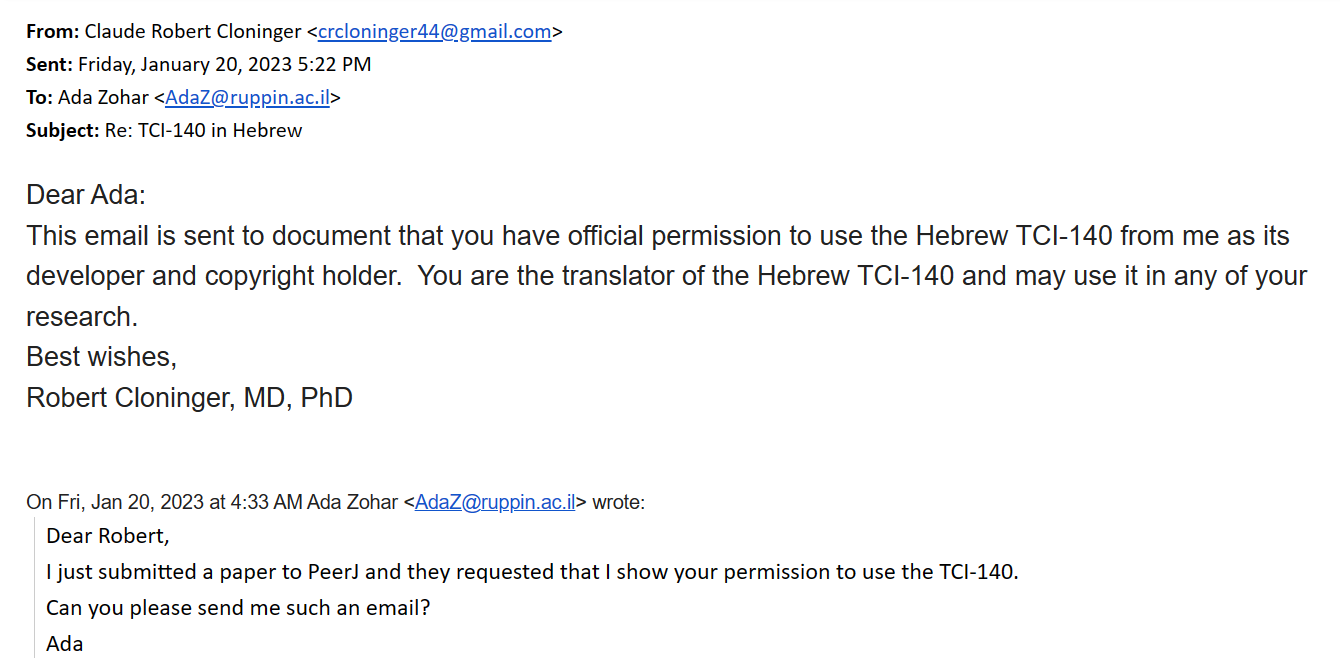


- SDQ

The use is in accordance with a published license: [The strengths and difficulties questionnaire (SDQ): Mentally Healthy Schools](https://mentallyhealthyschools.org.uk/resources/the-strengths-and-difficulties-questionnaire-sdq/)
